# Valorizing the usage of olive leaves, bioactive compounds, biological activities, and food applications: A comprehensive review

**DOI:** 10.3389/fnut.2022.1008349

**Published:** 2022-11-08

**Authors:** Samy Selim, Mha Albqmi, Mohammad M. Al-Sanea, Taghreed S. Alnusaire, Mohammed S. Almuhayawi, Hamada AbdElgawad, Soad K. Al Jaouni, Amr Elkelish, Shaimaa Hussein, Mona Warrad, Mohamed T. El-Saadony

**Affiliations:** ^1^Department of Clinical Laboratory Sciences, College of Applied Medical Sciences, Jouf University, Sakaka, Saudi Arabia; ^2^Olive Research Center, Jouf University, Sakaka, Saudi Arabia; ^3^Department of Chemistry, College of Science and Arts, Jouf University, Al Qurayyat, Saudi Arabia; ^4^Department of Pharmaceutical Chemistry, College of Pharmacy, Jouf University, Sakaka, Saudi Arabia; ^5^Department of Biology, College of Science, Jouf University, Sakaka, Saudi Arabia; ^6^Department of Medical Microbiology and Parasitology, Faculty of Medicine, King Abdulaziz University, Jeddah, Saudi Arabia; ^7^Department of Botany and Microbiology, Faculty of Science, Beni-Suef University, Beni-Suef, Egypt; ^8^Department of Hematology and Oncology, Yousef Abdulatif Jameel Scientific Chair of Prophetic Medicine Application, Faculty of Medicine, King Abdulaziz University, Jeddah, Saudi Arabia; ^9^Department of Botany and Microbiology, Faculty of Science, Suez Canal University, Ismailia, Egypt; ^10^Department of Pharmacology, College of Pharmacy, Jouf University, Sakaka, Saudi Arabia; ^11^Department of Clinical Laboratory Sciences, College of Applied Medical Sciences at Al-Quriat, Jouf University, Al Qurayyat, Saudi Arabia; ^12^Department of Agricultural Microbiology, Faculty of Agriculture, Zagazig University, Zagazig, Egypt

**Keywords:** olive oil products, antioxidant, antimicrobial, anticancer, food application

## Abstract

Olive oil production is a significant source of economic profit for Mediterranean nations, accounting for around 98 percent of global output. Olive oil usage has increased dramatically in recent years, owing to its organoleptic characteristics and rising knowledge of its health advantages. The culture of olive trees and the manufacture of industrial and table olive oil produces enormous volumes of solid waste and dark liquid effluents, involving olive leaves, pomace, and olive oil mill wastewaters. These by-products cause an economic issue for manufacturers and pose major environmental concerns. As a result, partial reuse, like other agronomical production wastes, is a goal to be achieved. Because these by-products are high in bioactive chemicals, which, if isolated, might denote components with significant added value for the food, cosmetic, and nutraceutical sectors, indeed, they include significant amounts of beneficial organic acids, carbohydrates, proteins, fibers, and phenolic materials, which are distributed differently between the various wastes depending on the olive oil production method and table olive agronomical techniques. However, the extraction and recovery of bioactive materials from chosen by-products is a significant problem of their reasonable value, and rigorous detection and quantification are required. The primary aims of this review in this context are to outline the vital bioactive chemicals in olive by-products, evaluate the main developments in extraction, purification, and identification, and study their uses in food packaging systems and safety problems.

## Introduction

*Olea europaea* L., an evergreen tree member of the Oleaceae family, occupying a total area of 10.8 million hectares, has been cultivated in 41 nations, particularly those in the Mediterranean basin (1). In 2018, the worldwide olive output was 21.6 million tons, and the global olive oil production was 3.2 million. With almost 23 percent of the world’s olive groves, Spain is the biggest producer of olive oil (1). Olives are utilized for oil extraction and, to a lesser extent, as table olives due to their extreme bitterness; nonetheless, they are rarely consumed as natural fruit. Olive oil is one of the oldest known vegetable oils and is commonly utilized in the Mediterranean diet (2) due to its outstanding sensory fragrance. The benefits of olive oil are attributable to various bioactive components, primarily monounsaturated and polyunsaturated fatty acids, squalene, triterpenic acids, phytosterols, alcohols, tocopherols, and polyphenols, which have powerful antioxidant activity (3). Oleuropein is the primary phenolic component in olive oil, which has powerful antioxidant activity and several pharmacological actions and therapeutic effects were observed (4).

Olive oil may be extracted using various methods, the most common of which are the classic pressing method, the two-phase decanter method, and the three-phase decanter method. In three-phase decanters, hot water is added to a process that dilutes water-soluble chemicals and separates the paste into three components: the oil phase, solid trash (pomace and stones), and vegetal water. One of the disadvantages of this method is that it generates a substantial amount of wastewater, which has a detrimental influence on the environment (5). The two-phase decanter method was created in the 1990s as an eco-friendly way to decrease oil mill waste (6). Despite olive oil’s economic and nutritional benefits, vast quantities of trash are produced in the cultivation fields and olive mills during olive oil manufacturing. Therefore, analyzing all possible circular economic paths in the olive oil supply chain is essential. Stempfle et al. (7) delineated these probable paths based on a thorough literature assessment. In this context, olive mill wastewater (OMWW) varies from 1.2 to 1.8 m^3^ per tons of olives (8). It is one of the major residues in the olive industry. This residue has significant environmental effects, including pollution of soil, emission of greenhouse gases, production of disagreeable scents, and suppression of plant and insect development (9). In addition, OMWW has low biodegradability, primarily attributable to its high organic load and chemical oxygen demand and the presence of organ halogenated pollutants, fatty acids, phenolic compounds, and tannins, which can limit the biological decomposition of OMWW (10). On the other hand, olive pomace is estimated at 15,655,000 tons worldwide each year (11), It is characterized by low pH, high organic pollutant load, and phytotoxic impact, which have become a significant environmental problem (12).

During olive oil extraction operations, various bioactive substances, mostly phenolic compounds, can be preserved in olive byproducts, particularly olive pomace, olive leaves, and OMWW. Olive byproducts are recognized as “raw materials” rather than “trash” due to their richness of nutrients, value-added additives, and bioactive chemicals, and significant research efforts have been focused on the recovery of phenolic compounds and terpenoids from olive byproducts. Green approaches for the recovery of bioactive compounds have recently been presented as a sustainable alternative to traditional procedures to safeguard human health, conserve the environment, and increase industry competitiveness by adopting a green strategy. Pressurized liquid extraction (PLE), ultrasonic-assisted extraction (UAE), enzyme-assisted extraction, microwave-assisted extraction (MAE), supercritical fluid extraction (SFE), molecular distillation, reduced-pressure extraction, and membrane separation technologies are some of these innovative techniques. For cosmetics (13), pharmaceuticals (14), food (15), and packaging applications (16).

The scientific community has paid special attention to utilizing olive wastes and byproducts because olive byproducts are rich in bioactive chemicals that can improve the functional qualities of packaging systems and lengthen the shelf life of food. Their use in food packaging has recently been researched. Despite a wealth of research on the extraction optimization and chemical characterization of active agents derived from olive oil byproducts, their impact on the functional qualities of food packaging materials remains obscure.

Olive leaves are one of the most important byproducts of olive farming, constituting 10% of the entire olive harvest weight and 25 kg/tree during olive tree pruning (17). Olive leaf extract (OLE) is one of the olive oil industry’s most valuable byproducts. It is a dark brown liquid with a bitter flavor and a large proportion of active chemicals belonging to secoiridoids, hydroxytyrosol, polyphenols, triterpenes, and flavonoids, which vary in concentration according to cultivar and environment (18). Oleuropein is a secoiridoid, more precisely the heterotic ester of elenolic acid and hydroxytyrosol, and is present in olive skin, seeds, oil, and notably leaves (19). Oleuropein is the primary molecule responsible for the OLE’s characteristics, including its powerful antioxidant and antibacterial activity (19).

Due to the high level of polyphenols (20), it has been utilized in traditional medicine for its multiple health advantages. Recent research has shown OLE’s potential use as a natural antibacterial agent (21), either used as an additive in foods (22) or, more typically, in food packaging, such as films to increase food safety (23). Antimicrobial composites can generate an environment that may delay or prevent the progression of microorganisms by decreasing the growth rate (24, 25). Natural antimicrobial agents extracted from plant material and wastes are more effective in health-related issues as many have gained GRAS (generally recognized as safe) status (26, 27). Compared to synthetic antioxidants and antimicrobial agents, this type of natural active packaging can preserve food for longer periods while posing no health risks (28–31). Due to the commercial availability of OLEs, more research has been conducted on the encapsulation of OLEs and encapsulation by other olive byproducts (32). Various wall components, including chitosan, maltodextrin, and polylactic acid, enclosed phenolics derived from olive byproducts (32). Consequently, the objectives of this review are to provide a synopsis of the principal bioactive compounds found in olive leaves, to examine the principal advancements in their extraction, purification, and characterization, and to discuss their application in food packaging safety issues.

## Olive (*Olea europaea*) morphology, origin and chemistry

Olive belongs to the family of Oleaceae, a small tree species native to the Mediterranean basin (33). Olives are one of the ancient plants grown for their fruits, and their aesthetic value was possibly a natural hybrid between *Olea laperinii* and *Olea ferruginea* (34). Crossing domesticated and wild olive varieties produces several new cultivars with superior fruit qualities to their parents; as a result, hundreds of variations are originated and propagated in various agro-climatic conditions worldwide (35, 36).

Olives have an annual growth habit and grow 3–12 m tall with 7.5 cm long leaves, which are lance-shaped and leathery with a dark green overhead and a silvery bottom color and are grown opposite each other on twigs. Mature olive fruits are smooth, edible, oval, and about 4 cm in size. Fruit size, color, shape, pit size, and surface morphology differ significantly between cultivars. Olives are mainly self-pollinated, but their production increases when cross-pollinated, mainly by the wind. During growth, the fruit color variations from lime green to purple, black, or brown.

The Mediterranean area is deemed the center of olive production and associated businesses. Apart from the Mediterranean area, the cultivation of olive trees is increasing in Afghanistan, India, Pakistan, USA, and Middle Eastern and African countries. The demand for this crop has increased with the growing concerns for health, research advancements, and awareness. Spain is not only the largest olive-producing country in the world but also the largest consumer. Total production in Spain was 4,577,800 from a cultured region of 2,500,000 ha (37).

In the USA, the native olive varieties are of better quality than others, so people trust the locally produced olive fruit and olive oil more. California *is becoming the Mediterranean* of the USA (38). In Pakistan, growers’ interest in olive cultivation is increasing due to the high global demand and rising prices in the international market. Pakistan has over 0.8 million hectares of wasteland suitable for olive farming. By covering this area with olive trees, Pakistan can produce about 1.84 million tons of olive oil; this could fetch over $6 billion. The presence of wild olive (Kahu) all around the Pothohar area indicated the possibility of successful cultivation and development of domesticated olive. The government of Punjab has already declared the Pothohar area as “Olive Valley.” About 2,500 olive varieties were discovered globally, out of which 250 are categorized as traditional olive cultivars by the International Olive Oil Council. However, classifying and identifying diverse olive varieties is challenging (38, 39). The production of olive oil was summarized in Table 1.^1^

**TABLE 1:** Worldwide production of olive oil.

Production (× 1000t)	2019/2020 (1)	2020/2021 (2)	% 2019/2020	Relative increase/Decrease	% World
World	3,266	3,010	−7.80%	−3.80%	100.00%
IOC members	3,078	2,810	−8.70%	−4.10%	93.30%
European union	1,920	2,051	6.80%	1.00%	68.10%
Spain	1,125	1,389	23.40%	1.60%	46.10%
Greece	275	275	0.00%	9.90%	9.10%
Italy	366	274	−25.40%	−5.00%	9.10%
Portugal	140	100	−28.80%	−10.10%	3.30%
Rest of EU	13	14	7.80%	−7.00%	0.50%
Other IOC countries	1,158	758	−34.50%	−15.60%	25.20%
Turkey	230	210	−8.70%	−2.80%	7.00%
Morocco	145	160	10.30%	7.60%	5.30%
Tunisia	440	140	−68.20%	−44.30%	4.70%
Algeria	126	70	−44.00%	−23.50%	2.30%
Argentina	30	30	0.00%	−5.50%	1.00%
Egypt	40	30	−25.00%	−20.30%	1.00%
Lebanon	14	26	85.70%	41.50%	0.90%
Jordan	34	24	−29.00%	1.60%	0.80%
Rest of IOC	99	68	−31.80%	−13.70%	2.20%
Non-IOC members	188	200	6.60%	1.10%	6.70%
Syria	118	115	−2.50%	−4.60%	3.80%
Australia	8	23	170.60%	30.50%	0.80%
Chile	20	20	2.50%	1.90%	0.70%
USA	16	16	0.00%	1.60%	0.50%
Rest non-IOC	26	26	2.00%	6.70%	0.90%

Olive fruit is composed of water (50%), oil (22%), cellulose (5.8%), carbohydrate (19.1%), protein (1.6%), phenolic content (1%), and inorganic materials (1.5%) (40). Other essential components include pigments, pectin, vitamins, and organic acids (38). Olive varieties with a lower oil content (<12%), such as Manzanilla, Ascolano, and Calamata, are used as table olives for pickle production. In contrast, those varieties that have a higher oil content (>20%), such as Picual, Arauco Nychati, Gemlik, Hojiblanca, Verdial, and Kalamonand, are preferred to be utilized for oil manufacture (41). In the case of table olive production, fruits with a larger size (>4 g) are preferred. In addition to fruit size, various other physical characteristics such as texture, flesh-to-pit ratio, color, and shape are crucial (42). Most of the varieties in Pakistan are Sevillano, Manzanilla, Gamlik, Nabali, Lccino, FS-17, Mission, Ascolano, BARI Zaitoon 1, and BARI Zaitoon 2.

## Olive products

**FIGURE 1:**
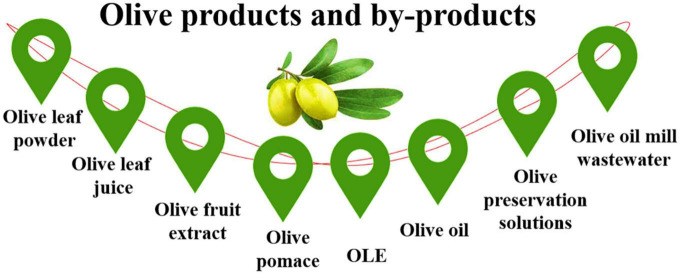
Different olive tree products and by-products.

The olive trees have been growing mainly for olive fruit, oil, leaf, and fine wood. The harvested olive fruits are mainly used in two forms, as table olive (10%) or made into oil (90%). In addition, olives have hundreds of commercially available food products. It is also used in cosmetics and health products. People take an extract from leaves and fruits to make medicine. The olive oil products and by-products are showed in Figure 1.

### Production of table olives

World consumption of table olives has undergone a constant increase. According to the International Olive Council (IOC), the major countries which produce table olives are Spain, Turkey, Egypt, Argentina, Morocco, Syria, Italy, Greece, and USA. Indeed, the largest consumers of table olives are also the greatest producers; in fact, the European Union (mainly Spain, Italy, Germany, and France) is being the area with the greatest consumption on a global scale followed by Turkey, USA, and Egypt (Table 2).

**TABLE 2:** Worldwide consumption of table olive.

Consumption (× 1000t)	2019/2020 (1)	2020/2021 (2)	Relative increase/Decrease	% Average	% World
World	3,268	3,125	−4.40%	2.90%	100.00%
IOC members	2,165	2,053	−5.20%	−2.00%	65.70%
European union	1,520	1,477	−2.90%	−2.00%	47.20%
Spain	522	538	3.10%	11.00%	17.20%
Italy	404	479	18.40%	2.10%	15.30%
France	130	128	−1.20%	7.60%	4.10%
Greece	115	112	−2.40%	−4.70%	3.60%
Germany	78	62	−19.70%	−5.50%	2.00%
Portugal	71	55	−22.50%	−19.60%	1.80%
Rest of EU	201	102	−49.00%	−43.70%	3.30%
Other IOC countries	645	576	−10.60%	−2.00%	18.40%
Turkey	170	160	−5.90%	−3.00%	5.10%
Morocco	140	140	0.00%	5.70%	4.50%
Algeria	115	80	−30.40%	−10.20%	2.60%
Egypt	43	30	−30.20%	−20.00%	1.00%
Tunisia	38	30	−21.10%	−13.70%	1.00%
Israel	28	25	−10.70%	5.30%	0.80%
Jordan	34	22	−36.80%	−10.40%	0.70%
Lebanon	8	20	166.70%	36.80%	0.60%
Rest of IOC	70	70	0.70%	4.10%	2.20%
Non-IOC members	1,104	1,072	−2.90%	13.90%	34.30%
USA	402	389	−3.40%	12.50%	12.40%
Other non-prod.	127	135	6.30%	63.10%	4.30%
Brazil	104	106	2.40%	30.70%	3.40%
Syria	104	86	−16.90%	−17.60%	2.80%
Japan	70	59	−15.10%	−5.00%	1.90%
Canada	58	58	0.90%	21.80%	1.90%
Australia	42	53	24.70%	14.30%	1.70%
China	58	53	−7.80%	7.60%	1.70%
Saudi Arabia	42	33	−20.50%	−6.00%	1.10%
Russia	27	32	18.50%	40.70%	1.00%
Mexico	17	18	2.90%	14.80%	0.60%
Switzerland	18	16	−13.90%	0.00%	0.50%
Rest non-IOC	36	34	−4.20%	5.70%	1.10%

The IOC has classified table olives into three groups: green olives, semi-ripe olives, and black or ripe olives (43). Overhead harvesters or tree shakers enhance the risk of bruising and result in soft fruits after processing, so mechanical vibrators or shakers have less application in producing table olives due to the potential risk of bruising (44). Worldwide, several methods are used for the processing of olives. Primary processing involves lye treatment, salt/heat drying, brining, soaking, and fermentation. In secondary processing, the olives’ organoleptic characteristics are enhanced by adding spices, almonds, cheeses, garlic, onion, herbs, vegetables, and stuffed with peppers. Globally, the most valuable table olive variations are Sevillano, Hojiblanca, Ascolana, Kalamata, Manzanillo, Tenera, and Conservolia (45, 46).

Olive shape, size, color, flesh-to-stone ratio, texture, and ease of destining are the essential characteristics of the selection of table olives (44). Raw olives are bitter due to the presence of oleuropein. So, just after harvesting, they are not suitable for consumption. Olives should be cured and fermented to make them palatable. The curing procedure may require a few days with lye or a few months with salt packing or brine. The flavor and taste of olive products depend on the fermentation, variety, olive oil, packing solutions, and processing solutions such as vinegar and flavorings (47).

The natural bitterness of olives should be removed by lye treatment. Lye should enter into the fruit to a depth of 2/3. Olives are dipped in 1.5–4.5% (w/v) NaOH solution at 15–25°C for 7–11 h, which varies according to environmental temperature and varieties. After that, the olives are removed from the tank and washed 4–5 times to remove alkali. Then olives are transferred to a fermentation tank with approximately 10% brine solution. The fermentation in olives is a lactic acid fermentation during which the sugars in olives are converted into lactic acid by lactic acid bacteria. Temperature, acidity level, pH, salt concentration, controlled microbial population, and flesh of the fruits are vitally important in the fermentation process. Fermentation takes 1–2 months, and the final processed table olives have a pH of 3.9–4, an acidity of 0.8–1% (lactic acid), and a 4–6% salt concentration (48).

Processing of table olives should also be done by oxidation of olives. Olives are dipped into a brine tank with a salt level of 5–6%, and the salt concentration depends on the olive and environmental conditions and varies up to 8–9%. The activity of unwanted microbes can be inhibited by adding 1.5–3% acetic acid, and a stable texture can be acquired by adding 0.1–0.3% CaCl_2_ (49). After being removed from the brine, olives are transferred to an oxidation tank to remove bitterness and darken the color through air, CO_2_, and lye treatment. Generally, 2–5 lime treatments are given depending on the ripeness, variety, environmental conditions, and penetration of lay-up to stone. Harvesting time is crucial for processing olives for a specific product (50).

Currently, the processor’s secondary processing of olives gains more attention, which includes incorporating numerous food products into olives to attain particular flavors. The different flavors are achieved by adding olive oil, vinegar, herbs, cheese, and spices (oregano, jalapenos, and garlic). Moreover, olives may be grouped with chili, garlic, peppers, fish, pimiento, cream, almonds, and many other fillers per the region’s taste (33). Vinegar is most commonly used for stuffing, which decreases pH and thus enhances shelf life. These stuffed olives are packed in vinegar, olive oil, brine, and sugar solution (51, 52). Numerous kinds of vinegar have commercial applications, such as wine, balsamic, or cider vinegar. For green olives, light-colored vinegar is preferred, while darker vinegar is preferred for black ones (53). Bitterness in olives is removed by bringing and then pitting.

### Olive oil processing

Inside the cells of olive fruits, the oil is partially located in the vacuole in a free form (approximately 76%), and the rest is found inside the cytoplasm, where it is dispersed as small droplets attached to colloids (54). Extra-virgin olive oil (EVOO) is obtained directly from olives, that is, pure olive juice. It is considered the highest quality oil, and in general, it is characterized for having a low acidity, up to 0.8% and a sensory grade higher than 6.5 points, thus, having perfect aroma and flavor. The conventional procedure for EVOO extraction includes a malaxation process, whose application increases yield compared to non-malaxated olives by approximately 5%, a significant improvement for the olive oil industries. However, the temperature and duration of malaxation can compromise the quality of olive oils. In the last decade, innovative mild techniques have been proposed to enhance EVOO production without a negative impact on the quality parameters (55).

Olive oil is obtained by pressing entire olive fruits and is generally used for cooking, pharmaceuticals, cosmetics, and soaps. Olive oil is used globally, but Mediterranean countries mainly accompany it.

#### Standard oil extraction

In old mills, olive oil extraction was done by batch procedures. In the original milling procedure, olives were crushed by two to six stone or granite wheels positioned in a big stone bowl, and the operation lasted 20–30 min. The olive paste was applied to disks made of hemp, coconut, and, more recently, synthetic fibers. The disks were stacked and inserted into the press. Since 1795, the disks’ hydraulic pressure has been used to compact the solid phase and expel the liquid phase of the olive paste, composed of oil and water (56). The classic extraction technique offers several advantages, including good extraction yields, the low moisture content in the oil, inexpensive equipment, and a small volume of wastewater created owing to the addition of a small amount of water. Nonetheless, it has additional drawbacks, including process discontinuity, poor capacity, and high labor expenses (57). Modern extraction technologies, including metallic crushers, malaxers, and centrifugal separation systems, have superseded conventional ones.

Modern oil extraction technologies are often practical and continuous. Presented here are the technologies now accessible for each unit operation, their accompanying processing factors, and their influence on olive oil quality.

#### Cleaning/Washing

The cleaning/washing process is the initial stage of olive treatment. Washing is essential for removing contaminants, particles, leaves, soil, stones, and dust, which impair olive oil quality and assure the mechanical safety of utilized equipment. Cleaning leaves and pollutants are accomplished by leaf collection and washing equipment. Olives are washed with recycled water and shaken in metal nets at the last stage of rinsing with clean water. It seeks to eliminate contaminants, such as pesticide residues. Removing pesticides from the olive surface before extraction is crucial, as most pesticides are oil-soluble and will transfer to the oil phase during extraction. The effect of washing on pesticide and herbicide residues is one of the knowledge gaps regarding olive oil quality. An intriguing investigation on the effect of washing in the presence of pesticide residues revealed that washing efficiently reduces herbicide concentration (58). In the same study, it was emphasized that the manner of olive harvesting was decisive: ground-harvested olives. The amounts of pesticides (mostly herbicides) were much greater in the washed than in the collected off the trees.

#### Olive grinding

Crushing the olives is a physical procedure that ruptures the olive fruit tissues and releases the oil droplets from the vacuoles of the plant cells. The proper equipment selection and crushing circumstances significantly impact the production yield and the quality of the olive oil.

#### Pre- or post-malaxation treatment procedures

Recent research has advocated the modification of malaxation equipment and the pre-treatment or post-treatment of the paste to increase the process’s efficiency. Paste pre-heating utilizing microwave (MW) therapy or high-power ultrasound or pulsed electric field (PEF) or flash thermal treatment application, or megasonic treatment, is one method for reducing the length or temperature of malaxation (59). One of the simplest pre-treatment methods involves heating or cooling to reduce malaxation time and the pace of chemical and enzymatic processes. The method of pre-malaxation employs tubular heat exchangers (60).

#### Malaxation

Malaxation is one of the most important phases in mechanical olive oil extraction since it impacts olive oil’s production, quality, and nutritional properties. Malaxation is the sole batch operation in the contemporary olive oil extraction process and happens after crushing. Malaxers are typically semicylindrical tanks with a shaft, rotating arms, and blades of various sizes and shapes. The tank features a heating jacket through which hot water flows to regulate the temperature. In most olive oil extraction methods, the malaxers are positioned horizontally, as is their shaft. In some extraction facilities, vertical malaxers (those with shorter length/depth parameters with a vertical shaft) are also utilized. During malaxation, the paste is stirred gently (usually 20–30 rpm) at moderate temperatures (often 20–35°C) for many minutes (30–45 min). As mentioned earlier, the oil droplets, freed from the oil cells during crushing, come into contact and coalesce (61). Malaxation and the subsequent coalescence are required for oil separation by physical separation techniques. Chemical compounds are transported concurrently between the solid, liquid, and oil phases with the necessary coalescence. In addition, chemical and enzymatic processes such as oxidation and hydrolysis influence olive oil’s chemical, nutritional, and sensory properties and the oil extraction yield (62).

Oil content depends on the variety, varying from 10 to 30% on a wet basis. In olive fruit, 96–98% oil is present in the pericarp and is mainly responsible for unique fragrance and flavor. Most of the fatty acids present in olive oil are gadoleic (C20:1), margaric (C17:0), Myristic (C14:0), oleic (C18:1), linoleic (C18:2), and linolenic (C18:3) stearic (C18:0), palmitoleic (C16:1) and palmitic acids (C16:0). Traces of eicosanoic acids are also present (63). Olive oil has 72–75% monounsaturated fatty acids, making it unique among all other seed oils because most are composed of polyunsaturated fatty acids (64). Olive oil has a long shelf life because of oleic acids, which make it less susceptible to oxidation and has antioxidant action and high stability (18).

## Olive by-products

Agricultural wastes pose the environment, despite they are rich in bioactive components (65, 66), therefore valorizing these wastes in many applications will maximize their usage in many applications (67, 68). Many by-products are produced during the processing of olives.

### Olive tree pruning biomass

Olive tree pruning, consisting of a woody fraction (25%), leaves (25%), and thin branches (50%), is one of the most plentiful and affordable lignocellulosic leftovers generated in Mediterranean nations. Olive tree pruning generated pulp and paper sheets (69), microfibrillated cellulose (70), and cellulose nanofibers. Abdel-Halim et al. (71) transformed cellulose isolated from olive tree pruning into water-soluble hydroxypropyl carboxymethyl cellulose using two etherification procedures. The cellulose had short fibers and low mechanical strength. Nanocellulose isolated from olive tree pruning using TEMPO-mediated oxidation pre-treatment followed by microfluidization exhibited a higher crystallinity index than eucalyptus nanocellulose, making it a novel candidate for reinforcing pulp, paper, and packaging films and coatings. The hydrophilic and semicrystalline structure of nanocellulose and the presence of lignin enhance its reinforcing effectiveness by establishing a dense network and giving enhanced packing functionalities to polymeric matrices (72). Manufacturing nanocellulose from plentiful and inexpensive olive byproducts for the food packaging business may be of tremendous importance. However, its extraction using intensive and time-consuming chemical methods that might generate substantial quantities of acidic wastewater is not commercially viable.

### Olive pomace

The solid phase remaining after olive oil extraction, olive pomace, consists of olive skin, stone, pulp, and kernel. This residue accounts for 35–40% of the total olive weight processed in the mill (73). Olive pomace consists of a lignocellulosic matrix, polyphenolic compounds, uronic acids, and fatty residues. Due to its abundance of antioxidant components such as polyphenols and carotenoids, olive pomace flour was added to chitosan-based forming solutions. However, the significant quantity of insoluble fibers in olive pomace flour might deleteriously affect the resultant films’ physical qualities. In order to address this issue, de Moraes Crizal et al. (74) created microparticles from olive pomace by spray drying and utilized them to create antioxidant packaging films. Olive pomace treated with saturated steam at 160°C was utilized to separate pectin fractions with commercially viable physical and biological qualities. Pectin can be a natural biopolymer for food packaging applications due to its film-forming, structural flexibility, gelling, emulsifying, and stabilizing capabilities. As food packaging materials, pectin-based films, emulsions, hydrogel beads, hydrogel coatings, aerogels, and nanoemulsions have been created. As wall materials, rocket seed gum and chia seed gum were used to encapsulate the active extract from olive pomace. The gum nanoparticles loaded with olive pomace extract demonstrated a high encapsulation efficiency, a delay in the release of the active extract at pH = 7.4, and improved antioxidant capacity (75). Nanoemulsion delivery technologies provide various advantages, including enhancements to the stability, efficiency, solubility, and controlled release of phenolic chemicals. Nanoencapsulation may be impractical due to its poor encapsulation effectiveness, usage of organic solvents, purification need, control of particle development, and difficulties in scalability (75).

### Olive mill wastewater

Olive mill wastewater, the aquatic waste produced by the three-phase olive oil manufacturing process, consists of 90 percent water and a negligible amount of organic compounds and mineral salts. It is anticipated that the yearly global output of OMWW ranges between 10 and 30 million m^3^ (76). Rich in phenolic chemicals (such as simple phenols, secoiridoid, flavonoids, and lignans) renowned for their powerful antioxidant potential and antibacterial activity, the OMWW phenolic fraction is desirable for the active packaging sector. Apicella et al. (77) evaluated the efficacy of various phenolic extracts obtained from OMWW and subjected them to various pre-treatments in developing multilayer antioxidant packaging. They discovered that the OMWW phenolic extract with the highest antioxidant activity and the lowest reducing sugar content was the most effective in developing antioxidant packaging due to its ease of incorporation into polylactic acid-based coating solutions. A novel OMWW derived from olive characteristics has more antioxidant activity than citrus pectins.

### Olive stones

Olive stones are composed mostly of cellulose (20.9%), hemicellulose (26%), xylose (26.6%), galactose (1.4%), arabinose (1.3%), and lignin (35.6%) and account for around 10% of the weight of olives (78). Adding olive stone flour as a reinforcing filler to plastics enhanced their flexural strength and water barrier qualities. In addition, olive stones are a viable source for the production of furfural, which may be employed as a crosslinking and reinforcing agent in packaging materials. In contrast, as part of the GO-OLIVA project, Spanish researchers have created Oliplast, a biodegradable packaging material made from olive stones that can be used to create trays, plates, and cups for olive oil containers (79).

### Olive cake

The olive skin, crushed pulp, and kernel shell that come after oil extraction still contain some oil, and about 25% of the water is called olive cake. The remaining first oil extraction from the olive by pressure is called simple olive cake. It has comparatively high oil (9%) and water (24%) content. It is tough to dispose of and store because air exposure rapidly oxidizes it (80). With the increasing trends in functional foods, reasonable practices should be adopted to separate valuable bioactive compounds from olive waste for its potential uses. The olive cake is cheap biomass in huge quantities, triggering many environmental problems in olive-growing countries (81). For this reason, olive cake is frequently used as fertilizer, fuel, and animal feed. Usually, in animal feed, the olive cake is mixed with molasses because of its lower palatability and used as a substitute for fiber because of its high cellulose content (82). Olive cake is an excellent source of phenolic and flavonoid content, so it has an extensive range of biological activities. Three features of antioxidant characteristics have been examined in olive cakes; antioxidant ability, free radical scavenging, and anti-radical (83).

Solvent extraction from the simple olive cake, usually by hexane, which contains comparatively less water and oil, is called exhausted olive cake (84). Tzamaloukas et al. (85) obtained a partly destoned olive cake by partially separating the shell from the pulp. They found that it has fewer portions of the shell, only those that cannot be detached from the pulp, which is why it contains less fiber and protein than simple olive cake. The olive cake is also called “fatty olive cake” if the cake is not being subjected to solvent extraction for oil separation (86). According to various investigations, olive cake contains phenolic compounds such as oleuropein (87, 88), caffeic acid and hydroxytyrosol catechol (81). Allouche et al. (89) identified tyrosol, rutin, vanillic acid, p-coumaric acid, verbascoside, and oleanolic acid. These bioactive compounds are potentially used for nutraceutical and pharmaceutical purposes (83). Some other researchers extracted different phenolic compounds from the olive cake using different solvents and concluded that the maximum antioxidant activity and total phenolic compounds were attained by using methanol at 70°C for 12 h. There is a slight difference in the quantities of the phenolic compounds (extracted by alkaline hydrolysis) among defatted and full-fat olive cakes, excluding quercetin and hesperidin, which are only observed in defatted olive cakes (88). There is a growing trend to discover the potential applications of this olive biomass in nutraceutical, pharmaceutical, and valuable food products to decrease environmental pollution and add value.

## Olive leaves

The growing olive trees and oil extraction produce substantial quantities of olive leaves throughout the year. About 25 kg of twigs and leaves are accumulated each year by pruning olive trees, and a large amount (about 10% of fruit weight) is separated from the fruits during oil extraction. Olive leaves are oblong, 5–10 cm long, 1–3 cm wide, and have a silvery green appearance. When consumed, these have a sharp, bitter taste (90, 91). Historically, olive leaves were generally used as a medicine to cure fever and other infections like malaria in the Mediterranean and European nations like Spain, Tunisia, Greece, Morocco, Italy, Turkey, Palestine, and France (42).

### Extraction of bioactive compounds of olive leaves

These leaves have been used as an extract or a whole host of powders. In some regions, these are consumed as olive tea, prepared with either fresh leaves or in dried form. Olive leaves comprise several potentially bioactive compounds with antimicrobial, antioxidant, hypoglycemic, anti-hypertensive, hypocholesterolemia, and anti-inflammatory properties (92). Several studies on animal models revealed that olive leaves could decrease arrhythmia, elevate blood flow, decrease blood pressure in the coronary arteries and avoid intestinal muscle spasms. Olive leaves also have antimicrobial properties against fungi, bacteria, and toxins (93, 94).

Each herbal substance has distinctive characteristics in extraction, so it is necessary to develop ideal circumstances for extraction. The extraction composition and assessment of its activities (antioxidant, antimicrobial) differ depending on different plant materials. The conventional extraction method is the most widely adopted method by researchers, in which soluble compounds are separated by diffusion from the solid phase (olive leaves) to a liquid matrix (solvent). This method is completed in two steps; first, the adsorption of solvent liquid into solid by capillary, osmotic forces an infusion of ions into the cells, followed by dispersion from the solid stage (95).

Extraction separates and assembles polyphenols and antioxidants from raw materials. The extraction procedure comprises a vigorous agitation of the ground leaves with some extraction solvent at elevated or ambient temperatures and consequent separation of the filtrate by filtration. Extraction can be repeated to improve extraction efficiency and yield (96). Many factors are involved in the effectiveness of the extraction procedure, like temperature, pH (97), solvent/solid ratio, solvent type (92) and particle size of the solid material, and the number of extraction steps (98, 99). Because of the different polarities of the solvent and extracted phenols, the solvent type strongly influences the extraction efficiency and antioxidant capacity of extracted phytochemicals (96).

**FIGURE 2:**
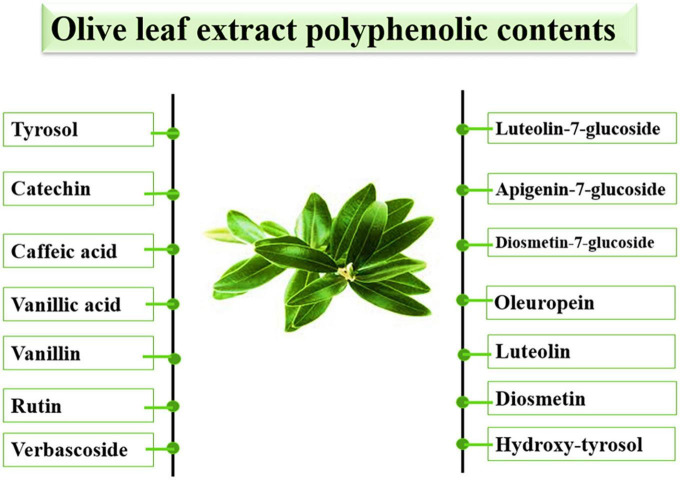
Different phenolic contents of olive leaves extract.

Solvent extraction is an inexpensive, efficient, and frequently applied procedure for food, pharmaceutical, and cosmetic purposes (100). However, extraction efficiency is affected by the chemical nature of the polyphenols to be extracted, various environmental and geographical influences, the extraction solvent, and future extraction use. Researchers have adopted many approaches to extract bioactive materials from olive leaves (Figure 2). The most important is extraction with super-heated liquids (101), followed by the conventional solvent extraction methods (Soxhlet extraction, maceration, hydro-distillation), microwave-assisted, ultrasound-assisted, and SFE (102).

Mylonaki et al. (103) investigated the extraction of phenolics from olive leaves by using 40, 50, and 60% ethanol (v/v) at pH 1, 3, and 5 for 1, 3, and 5 h, respectively. They concluded that a medium (50%) concentration of ethanol gave the lowest yield; in comparison, increasing or decreasing the concentration of ethanol from a medium favored the recovery of polyphenols in both cases. Some polyphenols of olive leaves are glycosides; it was estimated that their solubility was higher at 40% (v/v) ethanol (v/v) than at medium (50% v/v) ethanol concentration. Conversely, less polar phenolic compounds such as oleuropein increased yield in ethanol by 60% (v/v) than in (50% v/v). Researchers used boiling water, methanol, ethanol, diethyl ether, ethyl acetate, chloroform, butanol, and hexane as the foremost solvents used to extract olive leaves. Extraction of olive leaves with 80% methanol was stated as more efficient for the recovery of polyphenols as it gave a 95% recovery yield.

### Factors affecting the stability of phenolic materials during extraction

**FIGURE 3:**
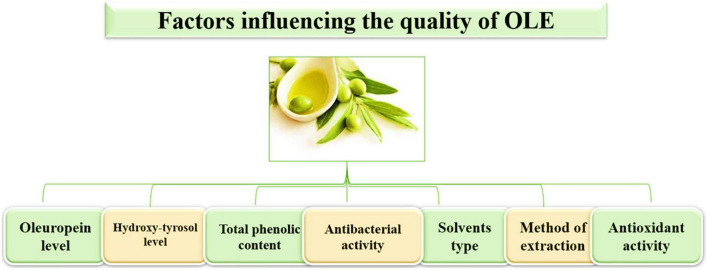
Factors influencing the quality of OLE.

There are different factors affecting the stability of phenolic compounds during extraction. Phenolic materials have polarity. Therefore, the highly polar organic solvent is more effective for extraction than non-polar ones. Methanol was considered a suitable solvent for the phenolic extraction of plants. However, it may cause unacceptable levels of toxic deposits in the extracts. For safety and many other reasons, water and ethanol are the most commonly used solvents (104). Various materials and solvents have historically been utilized as a foundation for extraction processes using leaves, and as seen in Figure 3, different factors influence the quality of OLE. Water has a critical function in extraction by facilitating the diffusion of extractable bioactive materials via plant cells. Sterile solutions (100%) of ethanol, methanol, and water were not as adequate as their binary solutions (combination of water and ethanol or methanol) for extracting phytochemicals from olives or other plant materials. The solubility of phenolic compounds could also be improved by altering the alcohol concentration, which might affect the extraction yield of polyphenols (105, 106).

#### Temperature

Bilek (107) studied the effects of solvent composition, temperature, duration, and solid-to-solvent ratio on the extraction of phenolic materials and interpreted the results using the response surface methodology. He used different concentrations of ethanol (20–100%) at variable temperature ranges (20–60°C) for 4–48 h, and the solid: the solvent ratio was from 4 to 8. It’s found that extraction time and solid-to-solvent ratio significantly affect phenolic compounds’ final yield. Similarly, Wu et al. (108) investigated the impact of temperature on antioxidant activity and phenolic concentration. They optimize temperature and solvent concentration conditions by using a central composite design. OLE was prepared at 20, 26, 40, 54, and 60°C and found that 40°C for ethanol extract gave 51 mg EAG g^–1^ and 50°C for methanol extract gave 56 mg EAG g^–1^ of polyphenol extraction yield and antioxidant activity for both was 90 and 92%, respectively.

#### Extraction time

Usually, less extraction time is required at high temperatures. Many studies have been done to prepare extract at room or slightly high at 40°C for 1 or 2 days under agitation temperature was maintained using a water bath (109, 110). Extraction by boiling water for 10–30 min was also reported (18, 91). This type of extraction is straightforward to operate and is a static process. The solid to solvent ratio is usually the sample weight (grams) and the volume of extracting solvent (milliliters). In the literature, the different ratios from 5 to 100 have been studied, but the ratio between 10 and 50 was mainly studied and considered as most suitable for extraction (111, 112).

#### Solvent-to-substrate ratio

Stamatopoulos et al. (112) used solid-liquid ratios of 1:5, 1:6, 1:7, 1:8, and 1:10 and discovered that increasing the solid-liquid ratio results in a decrease in the concentration of phenolic compounds. As the solid to liquid ratio increased, the solvent concentration increased, resulting in a diluted solution with a large yield of phenolic materials. Simultaneously, the concentration of compounds increased up to a ratio of 1:8 but remained constant above that (1:10). So, from an industrial and economic point of view, a solid to liquid ratio of 1:7 is best for extraction, which gives a high phenolic yield. The pH of the extraction solution is a critical issue because it defines the level of solubility for soluble bioactive materials and affects the degree of solubilization of hydrolyzable compounds (95).

#### pH

The pH up to 3.0 was adjusted with a 0.1 M KCl/HCl buffer solution, up to pH 6.5 with a 0.1 M sodium acetate/acetic acid buffer, and a pH of 8.3 was attained with a 0.1 M disodium hydrogen phosphate/HCl buffer system. The highest concentration of polyphenols was obtained at pH 1.3, indicating that pH significantly impacts polyphenols’ recovery. Solution pH mainly affects the solubility of active compounds in a solvent, with more excellent solubility resulting in greater yield in most instances. Similarly, Rodríguez-Juan et al. (113) had better extraction and antioxidant activity at pH 2.0 by preparing the extract with 60% ethanol for 5 h. They also concluded that increased pH values negatively impact the concentration of bioactive compounds obtained after extraction.

A comparison of yields obtained at pH 2.0 and pH 12.0 with pH 8.0 revealed that phenolic contents at pH 12 were decreased by up to 27% for apigenin-7-glucoside, 35% for luteolin-7-glucoside and oleuropein and 40% for verbascoside. The extraction yield at pH 2.0 for the target analyte was greater than pH 8.0. Solid phase extraction (SPE) and liquid-liquid extraction (LLE) are usually used for fractionation, refining, pre-concentration, and purification of phenolic materials. Purification of phenolics is based on their end users. Bouaziz and Sayadi (109) separated low molecular weight phenolic compounds using LLE. This crude OLE was vacuum dried to convert it into a concentrated form; then, the filtrate was dissolved in methanol and extracted with ethyl acetate. The process was repeated three times to obtain pure low molecular weight polyphenols (109).

#### Adsorbent

Moreover, silk fibroin was a new adsorbent technique to retrieve the required phenolic contents from OLE. Silk fibroin was mainly used as a promising adsorbent for separating rutin and oleuropein from OLE. The extract obtained from the extraction of olive leaves should be stored in conditions that result in minimum loss of activity of bioactive compounds and antioxidant effects. Light and temperature are the major factors that affect the antioxidant activity of polyphenols during storage. During storage, the destruction process was kept safe using an inert atmosphere (low in oxygen and high in nitrogen) and a dark room (114).

It was found that oleuropein remained stable in methanol extracts at room temperature for up to 30 days. After that, it would be degraded. Oleuropein remained stable for 7 days at room temperature in the water extract, completely lost its activity, and degraded after 17 days. It was recommended that liquid extracts of olive leaves be stored at a low temperature (0–40°C) in the absence of light, and powdered extracts can be stored under room conditions. Sometimes, antioxidants are added to extracts or the commercial capsuled OLE, with a shelf life of nearly 2 years under room conditions (95). Olive-leaf tea is soft and mellow, with many health benefits. Olive tea has been made thousands of years from olive leaves and is enjoyed all over the Mediterranean. Olive tea has a pleasant astringent flavor due to oleuropein, which is bitter. A potent infusion is required to capture olive leaves’ essence and herbal health benefits (101).

## Olive leaves extract active components and biological activities

Olive leaves are mainly used to make OLE, tea, powder, and capsules. The extract made from olive leaves is called “OLE.” Historically, OLE was utilized medicinally in different periods and places. Now, OLE is marketed as an antioxidant, anti-aging, cardioprotective, immune stimulator, antibiotic, anti-inflammatory, and blood sugar-regulating agent (115, 116). Olive leaves, primarily leaf extracts, are used in medicine, cosmetics, and as an additive in many food products. Different olive leaves extract active components, and their biological activities are mentioned in Table 3. The simpler one is the leaves’ infusion, which is used as a medicine. In this simple method, leaves are added to boiling water and infused for some time. A water extract of leaves is obtained (117).

**TABLE 3:** Phenolic compounds found in olive leaves extracts and their biological activities.

Phenolic compound	Biological activities	References
-Secoiridoids (oleuropein), -Simple phenols (Hydroxytyrosol, tyrosol), -Phenolic acids (Cinnamic acid, Gallic acid, Syringic acid, p-coumaric acid, Ferulic acid, Caffeic acid, Vanillic acid), -Flavonols (Rutin, Quercetin), -Flavanol (Catechin), and -Flavone (Luteolin)	Antioxidant activity	(210)
-Flavones (Luteolin, Luteolin-4′-O-glucoside, Apigenin, Apigenin-7-Oglucoside), and -Flavonols (Rutin, Quercetin)	Antioxidant and anticancer impacts	(211)
-Secoiridoids (Oleuropein), Phenolic acids (Verbascoside), -Flavones (Luteolin, glucoside)	Neuroprotective effects	(212)
-Secoiridoids (oleuropein, Oleacin, Oleuropein aglycone, Demethyloleuropein), and -Simple Phenols (Hydroxytyrosol, Tyrosol)	Not mentioned	(213)
-Secoiridoids (Oleuropein), and -Simple Phenols (Hydroxytyrosol)	Antioxidant effect	(16)
-Secoiridoids (Oleuropein, ligstroside, secologanoside) -Simple Phenols (hydroxytyrosol glucoside), -Flavones (luteolin glycosides), and -Phenolic acids (Verbascoside)	Antioxidant effects and cytoprotection in kidney cells	(214)
-Secoiridoids (Oleuropein), -Phenolic acids (Verbascoside), and -Flavones (Luteolin-4-O-glucoside, Luteolin-7-glucoside)	Antifungal effect	(215)
-Secoiridoids (Oleoside, Oleuropein, Oleuropein diglucoside, Oleuropein, aglycone), -Flavones (Apigenin, Luteolin 7-O-glucoside, Chrysoeriol-7-O-glucoside), -Flavonols (Rutin), -Phenolic acids (Verbascoside), -Simple phenols (Hydroxytyrosol), and other compounds (Elenolic acid glucoside)	Antioxidant and anti-asthmatic impacts	(4)
-Secoiridoids (Oleuropein)	Anticancer impact	(216)
-Secoiridoids (Oleuropein), -Simple Phenols (Tyrosol), -Phenolic acids (Vanillic acid, gallic acid), and -Flavanols (Catechin)	Antibacterial and anti-inflammatory impacts	(217)

There is increased concern about using natural resources for bioactive substances, food additives, and antioxidant compounds (118–121). It was found that a diet including a high proportion of fruits and vegetables is more effective against specific cancers and cardiovascular disorders (93, 122, 123). Many studies also support the concept that the ratio of certain tumors, like colon and breast tumors, is lower in Mediterranean regions where the daily diet contains olives and products (122). These impacts show that antioxidants, polyphenols, flavonoids, and vitamins are essential in disease prevention (119, 124–126). Finally, it was concluded that phenolics and flavonoids from olive leaves have many biological activities, so they also have some pharmacological actions or may have synergistic effects on those functions (92, 127, 128).

Although various groups of polyphenols and flavonoids have the antioxidant capacity, they generally have the o-dihydroxy (catechol) structure that confers the antioxidant activities to the OLE (93). However, the presence of phenolics is not the only factor considered for evaluating OLE antioxidant activity. The stability of the formed carboxyl group is another important factor that must be considered. The radical proxy species have electron delocalization ability, which is required for radical generation and stabilization. Phenolic extract from olive leaves can be used as an alternative to synthetic additives or antioxidants such as butylated hydroxytoluene (BHT) and butylated hydroxyanisole (BHA). The cheapest source of the phenolic extract is obtained from OMWW, which is further used as antioxidants in edible oils (129). Hayes et al. (130) found that OLE lowers lipid oxidation at 100 and 200 μg/g. This lowering effect is much better than that obtained from aerobic and modified atmosphere pack circumstances.

Similarly, caffeic acid is the most active natural antioxidant compared to synthetic ones (α-tocopherol BHA or BHT). In their study, Medina et al. (131) found that caffeic acid, even in small quantities in minced muscle meat, highly controls rancidity. In another study by Pazos et al. (132), glazing and spraying hydroxytyrosol on horse filets can reduce lipid oxidation. However, the spraying method showed better results due to more absorption and penetration of polyphenols by filets. Hydroxytyrosol at a level of 10–100 ppm can enhance the oxidative stability of fish oil, minced fish muscle, and oil-in-water emulsions. However, 50 ppm of hydroxytyrosol was more potent in diminishing lipid oxidation (132).

Also, OLE has antimicrobial activity against various microorganisms, such as; *Staphylococcus aureus*, *Salmonella typhi*, *Bacillus cereus*, *Escherichia coli*, *Vibrio parahaemolyticus*, and *Klebsiella pneumoniae* (133). Moreover, OLE modulates inflammatory and macrophage function responses, which might have activity against pathogens (134). Even though a single phenolic component of OLE may also have potent *in vitro* activities, the antimicrobial and antioxidant potential of the whole extract of the phenolic mix is better than that of individual phenolics (26).

It was also previously investigated by Pereira et al. (135) that extracts have been more advantageous than individual components since a bioactive compound can alter its potential in other extracted substances. OLE concentrations against *B. cereus* > *E. coli* > *S. aureus* > *Pseudomonas aeruginosa* > *Bacillus subtilis*. Markin et al. (133) stated that aqueous OLE at a level of 0.6% (w/v) could inhibit *K. pneumoniae*, *E. coli*, *S. aureus*, and *P. aeruginosa* in 3 h contact. *B. subtilis* died at a concentration of 20% (w/v) due to its spore-forming capacity. Results revealed that extract did not have broad-spectrum antibacterial potential but had a significant effect against *Campylobacter jejuni* and *Helicobacter pylori*. Ahmed et al. (19) studied the antibacterial effect of OLE on raw undeveined shrimp (*Penaeus semisulcatus*). OLE of different levels [0.5, 1, and 2% (w/v)] were prepared. At OLE 2%, *E. coli*, *V. parahaemolyticus*, *B. cereus*, *S. aureus*, *S. typhi*, and *K. pneumoniae*.

In addition, Aliabadi et al. (136) investigated the antimicrobial activity of some pathogenic bacteria such as *Salmonella typhimurium* PTCC 1639, *K. pneumoniae* PTCC 1053, *E. coli* PTCC 1399, *B. cereus* PTCC1274, and *S. aureus* PTCC 1431 by using the Agar well diffusion method. The results obtained showed that the extract has very high antimicrobial activity and maximum inhibition of 11.5 mm against *S. typhimurium* PTCC 1639. Moreover, Dogan et al. (137) prepared nanofibers using OLE and found their effectiveness as an antimicrobial agent. OLE-containing nanofibers have more antibacterial activities than antifungal potential. The nanofibrils, stored in OLE for a month, still have bioactivity after releasing 70–95% of the OLE from nanofibers.

Gokmen et al. (138) evaluated the antimicrobial activity of the OLE against ten bacteria by microdilution and disk diffusion; they found the inhibition zone of 13.33 + 2.08 mm and 21.67 + 1.53 mm was observed against *S. typhimurium* and *B. cereus*, respectively. The MICs for *E. coli* O157, *P. aeruginosa*, *Listeria monocytogenes*, and *Enterobacter sakazakii* were 32 mg mL^–1^, and the MICs for *S. aureus*, *E. coli*, *B. cereus*, *Enterococcus faecalis*, *Proteus vulgaris*, and *S. typhimurium* were 16 mg mL^–1^ by microdilution and disk diffusion. The inhibition zone of 13.33 + 2.08 mm and 21.67 + 1.53 mm was observed against *S. typhimurium* and *B. cereus*, respectively. The MICs for *E. coli* O157, *P. aeruginosa*, *L. monocytogenes*, and *E. sakazakii* were 32 mg mL^–1^, and the MICs for *S. aureus*, *E. coli*, *B. cereus*, *E. faecalis*, *P. vulgaris*, and *S. typhimurium* were 16 mg mL^–1^.

Furthermore, OLE can inhibit a broad spectrum of bacteria, viruses, yeast, and *Plasmodium falciparum*, the causative agent of malaria (137). Also, it can be used against a large spectrum of pathogens, involving *S. typhimurium*, *Erwinia carotovora*, *B. subtilis*, *E. coli*, *S. aureus*, *P. falciparum*, *Pseudomonas fluorescens*, *Candida albicans*, *B. cereus*, and *Corynebacterium* species (139, 140). Some pathogens can adulterate food surfaces post-processing, resulting in food with a reduced shelf life and increasing the possibility of foodborne infections. Food packaging ingredients can be mixed with antimicrobial additives to overcome the issue of pathogens during the distribution and storage of some food materials (28, 137).

Olive leaf extract activity is attributed to their polyphenols content (141). The combination of phenolic materials has a more beneficial effect than the individual one (26). Due to these activities and bioactive compounds, OLE and whole leaf usage have expanded in pharmaceutical, cosmetic, and food productions as functional food materials and additives (42).

The hot water extract of OLE yielded 94% of active compounds such as oleuropein and verbascoside (91). Japon-Lujan and Luque de Castro (102) investigated that 80% aqueous ethanol (v/v) was the best solvent for phenolic extraction from olive leaves, and it can be used as a replacement for toxic solvents (chloroform, diethyl ether, and methanol) to obtain phytochemicals for human consumption. Lee and Lee (26) also stated that total phenolic and flavonoid contents were considerably higher in the 80% ethyl acetate, butanol, and ethanol (v/v) extracts than in water, hexane, and chloroform extracts. When using ethanol as a solvent, some precautions must be taken into consideration; for example, a mixture of water and ethanol is exothermic, so have less volume than the separate sum of individual constituents. Ethanol solution over 50% (v/v) are flammable. It was also reported that ethyl acetate extract from olive leaves has a strong antioxidant capacity because ethyl acetate is less polar, giving a more active extract than ethanol and methanol (104).

Stamatopoulos et al. (112) extracted the dried olive leaves of 1 mm particle size with 20% ethanol 1:10 solid to solvent ratio at various pH 1.3, 2.0, 3.0, 4.2, 5.2, 6.5, and 8.3. Furthermore, Yateem et al. (142) also stated that total phenolic and flavonoid contents were considerably higher in the 80% ethyl acetate, butanol, and ethanol (v/v) extracts than in water, hexane, and chloroform extracts. When using ethanol as a solvent, some precautions must be taken into consideration; for example, a mixture of water and ethanol is exothermic, so it has less volume than the separate sum of individual constituents. Ethanol solutions over 50% (v/v) are flammable. It was also reported that ethyl acetate extract from olive leaves has a strong antioxidant capacity because ethyl acetate is less polar, giving a more active extract than ethanol and methanol.

Similarly, De Leonardis et al. (143) produce a pure OLE with high hydroxytyrosol content. For this purpose, they developed a procedure in which olive leaves are exposed to acid steam cooking that hydrolyzes the complex phenolic contents into simpler ones, so the required hydroxytyrosol was extracted with ethyl acetate by applying LLE (143). An antioxidant can be added to the extract to avoid the oxidation of phenolic compounds (95). Different fractions of flavonoids and phenolic acids were obtained from crude OLE by SPE using C_18_ cartridges. It was recommended to use pack C_18_ to extract crude olive leave extract to obtain pure polyphenols. Romani et al. (144) extracted olive leaves with diatomaceous earth with different eluents using solid-liquid extraction (SLE) techniques and got good separation peaks. This extraction method could quantify and detect several phenolic compounds (144).

## Food application of olive leaves extract

**FIGURE 4:**
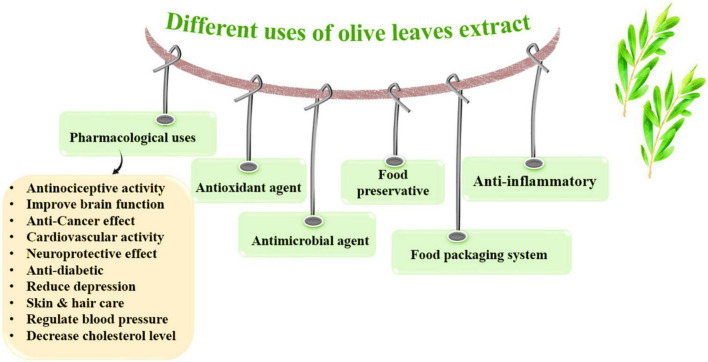
Different applications and benefits of olive leaves extract.

The benefits and applications of OLE and olive oil are summarized in Figure 4, and Table 4.

**TABLE 4:** Different uses of olive leaf extract in food processing and preservation.

Food	Used olive part	Impact of usage	References
Different refined oils	Methanol OLE extract	Olive leaves extract (OLE) addition substantially improved antioxidant capacity and oxidative stability for all the oils studied after supplementation	(218)
Refined olive oil	Hydroxytyrosol and other antioxidants	The addition of antioxidants (OLE) prevents the formation of toxic products like cholesterol oxides and increases their nutritional value	(219)
Sunflower oil	Microwave OLE and methanol extract	The OLE blocked the oxidation process while heating the oil at 70°C. Methanol extract gave better results.	(220)
Soybean oil	Nano-encapsulated OLE	Soybean oils had more oxidative stability when nano-encapsulated OLE was added.	(148)
Extra virgin olive oil	OLE methanol extract	Adding OLE to olive oil improves its nutritional value and antioxidant stability. That proved that adding OLE to a standard olive oil makes it similar to high-quality olive oil.	(148)
Table olives	Water OLE extract	Sensory evaluation of treated table olives showed an increase in bitterness	(221)
Apple	OLE (Ethanolic extract)	They sprayed a chitosan coating solution with OLE on apples. They found that “the addition of OLE to chitosan coating films meaningfully reduced the gradual decline in total phenolic, flavonoids and antioxidants.”	(222)
Milk	OLE Water extract	Addition of OLE to pasteurized milk. The results have been excellent, as OLE was effective against *Bacillus cereus* and inhibited the enzyme α-glucosidase, which is related to the degradation of milk.	(223)
Tomato paste	Encapsulated OLE. Methanol/Water extract	Encapsulated OLE in tomato paste compared to non-encapsulated ones have better results.	(224)
Backed snacks	Water extract	OLE reduced lipid oxidation increasing its shelf life consequently. The addition of OLE is highly recommended when low-quality olive oil is used.	(225)
Beef cubes	OLE	A study immersing beef cubes in an OLE solution found that “using 2 and 3% OLE had the beneficial effect in controlling the microbial load of beef cubes during 9 days of storage at 4°C.”	(226)
Salmon burgers	Hydrolyzed OLE and OLE	OLE decreased lipids oxidation in salmon. Salmon mince treated with hydrolyzed OLE showed lower microbial counts than OLE during the study, which extended the shelf life of the fish product to 4°C and vacuum-packed.	(227)
Shrimp	OLE (Ethanol extract)	OLE reduced the count of the aerobic and coliforms bacteria by at least 1 log cycle colonies forming unit (CFU) g^–1^ in comparison to the non-treated control group by immersing shrimp in a solution containing OLE at a concentration of 1% (w/v).	(19)
Smoked salmon	Olive leaf powder (OLP) and water ethanol extract (OLE)	OLE was tested against *L. monocytogenes* in a food matrix well known as being a food that can be infected by *L. monocytogenes*, which is the cold-smoked salmon. Edible fish gelatin films with OLE showed antibacterial activity against *L. monocytogenes* in agar diffusion tests and the fish over storage. They found that de optimal film formulation is 5.63% OLE to be effective against *L. monocytogenes*	(228)
Minced beef	Irradiated OLE	Test of OLE in minced beef focused more on the antibacterial activity with important activity from the microbiological against *B. cereus*, *S. aureus*, *E. coli*, *K. pneumoniae*, *P. aeruginosa*, and *S. typhimurium* “The results indicated that when 2, and 3 ml OLE were added to 100 mg of minced beef could improve quality attributes and extend shelf life of minced beef from 1 week to 3 and 4 weeks under cold storage.”	(229)
Lamb meat patties	Olive waste extract	Similar results to other studies concluding that “This application reduced lipid and protein oxidation while maintaining an acceptable color for a longer period, representing a 3-day in the shelf-life extension compared to patties without the added extract.”	(230)
Frozen hamburger	Pure oleuropein	This study added a specific concentration of oleuropein to frozen burgers and compared it to the food additive sodium erythorbate to compare their antioxidant activity. In this test, both antioxidants had similar results suggesting that oleuropein is a potential substitute for synthetic antioxidants in food like sodium erythorbate.	(231)
Frankfurt sausages	OLE (Ethanol extract)	OLE in frankfurter-type sausages showed that OLE has antioxidant activity and significantly reduces the total count of bacteria in a 45 days study at 4°C.	(232)
Anchovies	Lyophilized methanol extract	OLE does not change organoleptic properties and is effective against spoilage bacteria for 22 days.	(233)
Strawberry	OLE (Ethanol extract)	The experiment was done by spraying different coating formulas. They found that chitosan coating with OLE “led to keep the bioactive substances of cold-stored strawberry fruits.”	(234)
Minced meat	Olive leaf powder	Olive leaf powder added to minced beef significantly reduced bacteria (mesophilic aerobic bacteria) in meat products. In this study, they found that olive leaf powder has an instantaneous antimicrobial effect and controls the microbial count for 6 days at 4°C.	(235)
Fresh hamburger	OLE and oleuropein	0.5% of oleuropein and 1.5% of OLE were considered adequate to preserve fresh hamburger.	(236)
Lard	Olive-oil mill wastewater	Olive mill wastewater is highly effective for oxidative stabilization of lard also at low temperatures	(237)
Yogurt	Olive pomace	Olive pomace made the yogurt fulfill the nutritional claim of being a “source of fiber,” improved the nutritional value of the yogurt, and made it more stable from the nutritional point of view.	(238)
Gluten-free bread	Water extract	The addition of OLE and lactic acid bacteria (LAB) in gluten-free bread improved sensorial properties and reduced the fungi count, increasing consequently its shelf life	(239)

### Antioxidant agent

Synthetic antioxidants and antimicrobials are harmful to human health. For instance, BHT usage in rat feed causes fatal hemorrhages in the pleural and peritoneal cavities and organs, including the pancreas and epididymis testes. This outcome is attributable to BHT’s capacity to reduce blood coagulation factors dependent on vitamin K (145). In contrast, BHA has come under fire for its potential to promote carcinogenesis and create lesions in the front stomach of rats (146). Synthetic antibacterial and antioxidant compounds have been widely employed in food and cosmetics to inhibit lipid oxidation. Currently, synthetic antioxidants are less popular due to consumers’ growing awareness of the adverse effects of chemical additions (147). As a result, there is a compelling case for using phytochemicals, especially essential oils, flavonoids, and polyphenols extracted from olive leaves and fruits, as an alternative to synthetic additions (146). Phenolic extract from olive leaves can be used as an alternative to synthetic additives or antioxidants such as BHT and BHA. The cheapest source of the phenolic extract is obtained from OMWW that is further used as antioxidants in edible oils (129). Hayes et al. (130) found that OLE lowers lipid oxidation at 100 and 200 μg/g. This lowering effect is much better than that obtained from aerobic and modified atmosphere pack conditions. Similarly, caffeic acid was the most active natural antioxidant compared to synthetic ones (α-tocopherol BHA or BHT).

Medina et al. (131) studied that caffeic acid, even in small quantities in minced muscle meat, has a high control of rancidity. In another study by Pazos et al. (132) glazing and spraying hydroxytyrosol on horse filets can reduce lipid oxidation. However, it was found that the spraying method showed better results due to more absorption and penetration of polyphenols by filets. Hydroxytyrosol at a concentration of 10–100 ppm can enhance the oxidative stability of fish oil, minced fish muscle, and oil-in-water emulsions. However, a 50 ppm level of hydroxytyrosol was more effective in minimizing lipid oxidation (132).

The significance of olive leaves as industrial and agricultural waste is emphasized by explaining their availability, therapeutic and nutritional effects, and research in this field. Additionally, it highlights the role of OLE as an antioxidant and presents methods for determining its antioxidant potential. Furthermore, it provides an overview of the presence of enzymes. Mohammadi et al. (148) investigated the antioxidant activity of OLE encapsulated by nano-emulsions to preserve soybean oil. The average droplet size 1 day after production was 6.16 nm for the primary W/O nano-emulsion and 675 nm and 1,443 nm for multiple emulsions stabilized by WPC alone and a complex of WPC–pectin, respectively. The antioxidant activity of these emulsions containing three concentrations of 100, 200, and 300 mg OLE during storage was evaluated in soybean oil by peroxide value, TBA value, and rancimat thermal stability test and was compared with blank (non-encapsulated) OLE and synthetic TBHQ antioxidant. Nano-encapsulated OLE was capable of controlling peroxide value better than unencapsulated OLE. However, because of the blocking of phenolic compounds within dispersed emulsions droplets, the thermal stability of encapsulated OLE was lower.

Also, González-Ortega et al. (149) examined the inhibitory effect of oleuropein against lipid peroxidation of DPPC liposomes. In model and commercial beverages, soy phosphatidylcholine (PL-90 g) was encapsulated in soy phosphatidylcholine (PL-90 g). Oleuropein generated a widening and splitting of the gel-to-liquid phase transition temperature that was concentration-dependent. Fluorescence tests demonstrated a fluidizing impact on liposomes below their gel-to-liquid phase transition temperature and a more significant lipid ordering above, particularly concerning active encapsulation. In PL-90 g liposomes, oleuropein demonstrated an antioxidant activity against lipid peroxidation. In conclusion, more significant effects were observed on the structure and fluidity of DPPC liposomes when oleuropein was actively encapsulated. In contrast, its incorporation into acidic foods in encapsulated form could enhance its stability.

### Antimicrobial agent

Presently, the resistance of microorganisms to antibiotics is more significant than ever. Therefore, there are numerous recent papers about alternative solutions for inhibiting their influence on human health. Olive leaf is studied as an essential source of antimicrobials with a low cost and is used in medicine. Numerous publications involving green technologies for isolating active compounds from olive leaves have appeared over the past few decades. Borjan et al. (150) stated that phenolic compounds isolated from olive leaves have suitable biological activities, especially antimicrobial. This paper uses recent research findings with a wide range of study models to describe the antimicrobial potential of phenolic compounds. It also describes the vast range of information about methods for the determination of antimicrobial potential, focusing on effects on different microbes.

Olive leaf extract, being a natural substance, has the potential to be utilized to combat foodborne bacteria as an antibiotic. Liu et al. (151) studied the antibacterial activity of OLE against significant foodborne pathogens, such as *L. monocytogenes*, *E. coli* O157:H7, and *Salmonella enteritidis*. Our results showed that at a dose of 62.5 mg/ml, OLE suppressed the development of these three pathogens virtually entirely. In addition, OLE inhibited the cell motility of *L. monocytogenes*, which corresponded with the lack of flagella, as seen by scanning electron microscopy. In addition, OLE reduced biofilm production by *L. monocytogenes* and *S. enteritidis*.

In the investigation of Topuz and Bayram (152), they isolated oleuropein from the leaves of various olive varietals and evaluated its antioxidant and antibacterial properties. The quantity of oleuropein in olive leaf products ranged from 215.26 to 958.22 mg/g. The antioxidant activity of olive leaf products ranged between 104.83 mg TE/g (ABTS) and 456.93 mg TE/g (DPPH), respectively. On the other hand, MIC values varied between 50 and 0.78 mg/ml for investigated microorganism cultures. *S. aureus* is the most susceptible bacteria to oleuropein extracts, whereas *E. coli* O157:H7 is the most resistant. Due to their proven antioxidant and antibacterial capabilities, CEs and oleuropein extracted from these CEs can potentially increase the shelf life of food goods.

Food spoilage due to microorganisms has been a main worldwide issue for many years, and still, a large quantity of food has become unusable due to spoilage. Some plant extracts can use as antioxidants and antimicrobials in food systems. These natural products have a critical role in food preservation and extending shelf life without harming consumers (153). Food resources’ microbiological safety and shelf life are strongly correlated. Despite being stored in a refrigerator, food material must have its shelf-life reduced owing to microbial deterioration. Antimicrobial agents should be used to rectify the safety and hygiene problems that occur when food is processed and stored (154).

**FIGURE 5:**
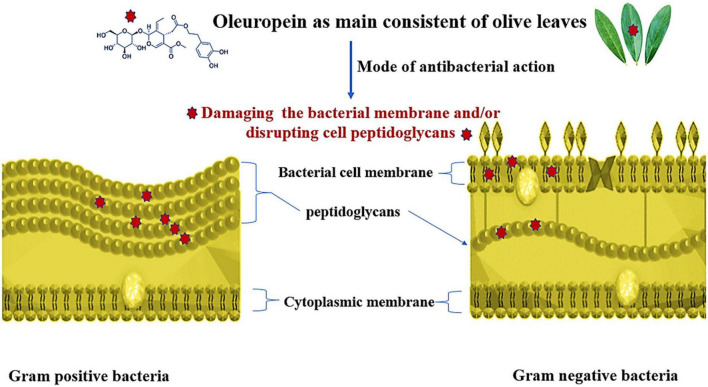
Mode of antibacterial action of oleuropein as main component of olive leaves; oleuropein inhibit both Gram negative and Gram positive bacteria propagation via damaging the bacterial membrane and/or disrupting cell peptidoglycans.

Many Gram-negative and gram-positive bacteria, yeast, and parasites, including *P. falciparum*, which causes malaria, are inhibited by olive (leaf and fruit) extract. Renis (155) it has been proven that olive leaves contain elenolic acid and its salts, calcium elenolate, which are effective against several viruses, including the myxoviruses that cause pseudorabies, leukemia virus, and encephalomyocarditis, as well as the polioviruses 1, 2, and 3, and the rhinoviruses. The effectiveness of OLE against various pathogens and food spoilage agents, including *B. subtilis*, *E. carotovora*, *S. typhimurium*, *P. falciparum*, *E. coli*, *S. aureus*, *B. cereus*, *C. albicans*, *P. fluorescens*, and *Corynebacterium* sp. (156). The antimicrobial impacts of OLE were summarized in Table 5 and the antibacterial mode of oleuropein action against both Gram positive and Gram-negative bacteria as main consistent of olive leaves was presented in Figure 5.

**TABLE 5:** Antimicrobial activity of olive leaves extracts.

Antimicrobial activity	Microorganism	References
**Antibacterial**	*Klebsiella pneumoniae*, *Escherichia coli*, and *B. cereus*	(240)
	*L. monocytogenes*, *E. coli* O157:H7 and *S. enteritidis*	(151)
	*Escherichia coli*, *Staphylococcus aureus*, *Klebsiella pneumoniae*, *Bacillus cereus*, *Salmonella typhi*, and *Vibrio parahaemolyticus*	(19)
	*Mycoplasma*	(94)
	*Staphylococcus aureus*, *Salmonella enteritidis*, and *Bacillus cereus*	(241)
	*Escherichia coli*, *Pseudomonas aeruginosa*, *Staphylococcus aureus*, *Bacillus subtilis*, and *Klebsiella pneumoniae*, *Campylobacter jejuni*, *Helicobacter pylori*, and *methicillin-resistant Staphylococcus aureus*	(133)
**Antiviral**	Viral hemorrhagic septicemia rhabdovirus	(242)
	White spot virus syndrome	(243)
	Hepatitis B virus	(244)
	Herpes simplex virus	(245)
	Human immunodeficiency virus (HIV)	(134)
	Herpes simplex virus type 1	(246)
	Influenza virus	(247)
**Antifungal**	*Candida albicans* and *Cryptococcus neoformans* (fungi)	(135)
	*Candida albicans*, *Candida glabrata*, and *Candida parapsilosis*	(133)
	*Aspergillus flavus*	(248)
	Dermatophytes–*Trichophyton mentagrophytes*, *Microsporum canis*, and *Trichophyton rubrum*; *Candida albicans*	(133)
**Antiparasitic**	*Giardia lamblia* cysts	(249)
	*Leishmania*	(250, 251)
	*Limnatis nilotica*	(252)
	*Coccidia*	(253)
	*Schistosoma mansoni*	(254)
	*Plasmodium falciparum*	(255)
	*Cryptosporidium*	(255, 256)

### In the food packaging system

Food surfaces could be adulterated during post-processing by pathogens. The shelf stability of food is reduced while the danger of foodborne infection elevates. Antimicrobial agents can be added directly to the food surfaces, but these agents migrate rapidly into the food or become neutralized, decreasing their benefits. An alternate method of using an antibacterial chemical for food safety is antimicrobial packaging (157). Food packaging materials can be mixed with antimicrobial components or additives to overcome this major issue of pathogens during the distribution and storage of some food products (28, 137).

Active packaging films are mainly used for preserving liquid foods, such as egg whites and fruit juices, and for the surface preservation of solid foods. When the packaging material undergoes some activity, the packaging sheet prevents microbes by lowering the growth rate or reducing the live counts of microbes. Antimicrobial activity in the packaging system can be achieved with natural and chemical antimicrobial agents (157). Antimicrobial composites may delay or prevent the progression of microorganisms by decreasing the growth rate and prolonging the lag phase (19, 158). Natural antimicrobial agents extracted from plant material are more effective in health relating issues because most of them have gained GRAS (generally recognized as safe) status (26). This active packaging can preserve food without any health hazards due to synthetic antioxidants and antimicrobial agents (28, 30).

Olive has several bioactive polyphenols that inhibit the microorganisms’ growth by inhibiting specific enzymes in the membrane, which destroy cell membranes (159). Antimicrobial compounds can be combined with food packaging materials to overcome the problem of unwanted microorganisms in some food products during distribution and storage (28). Antimicrobial compounds inside the packaging can generate an environment that may prevent or delay the growth of microorganisms by reducing the growth rate and extending the lag period. Antimicrobial packaging films were applied for surface preservation, and liquid foods such as fruit juice or egg white were preserved in antimicrobial utensils and containers (28, 30).

Much research studies the effectiveness of the active packaging on food spoilage organisms such as *E. coli*, *S. aureus*, *S. enteritidis*, *L. monocytogenes*, and *S. typhimurium* (160). Turhan (30) incorporates OLE into methylcellulose, PLA (an aliphatic polyester prepared by lactic acid) building blocks, and MC-PLA films and checks its effectiveness against *S. aureus*. In another research, Ayana and Turhan (161) used OLE-containing MC films to pack kasar cheese inoculated with *S. aureus* and examined the inhibition effect of the films. The water activity that determines the quality and storage of food products affects the packaging material’s water vapor permeability (WVP). As a result, mechanical characteristics and WVP were considered to assess the efficiency of packaging sensorial attributes. It was observed that the maximum mechanical properties and minimum WVP were in MC films that had 1.5 and 2.0 g OLE/100 mL film solution, respectively. However, increasing the OLE level increased the film’s antimicrobial activity and bitterness while decreasing the packaging film’s transparency. Thus, 1.5 g of OLE-containing packaging film has more inhibition zone for *S. aureus*, and their numbers were lowered by a 1.22 log cycle.

## Pharmacological importance of olive leave extract

### Antinociceptive activity

The management of pain is a leading clinical dilemma. Some medicines (Opioids) are generally used to treat moderate to severe pain, but these drugs have numerous side effects, like physical dependence, drowsiness, constipation, emesis, and analgesic tolerance. Thus, efforts are made to manage pain by finding herbs without hazardous side effects. Olive oil has been used to relieve joint and muscle pain in Iran and to cure neuralgia and rheumatic diseases in some regions of Lebanon (162). In conventional medicine, OLE was used as an anti-inflammatory, vasodilatory, antinociceptive, hypotensive, anti-rheumatic, hypoglycemic, antidiuretic, and antipyretic agent. It is also studied that Ca^2+^ can regulate pain sensitivity, and inhibiting the movement of calcium causes antinociception (154, 163).

Additionally, L-type Ca^2+^ channels have antagonists’ effect of producing numbness after central and peripheral administration (164). It is the fact that Ca^2+^ influx is crucial for pain sensitivity, and its blockage is necessary for revealing pain; OLE might exert antinociceptive effects on thermal and chemical models of pain (162, 165). The prevalence of diabetes mellitus has increased because of poor diet, sedentary behavior, population expansion, obesity, and aging. It is acknowledged that hyperglycemia, or elevated blood sugar, is a major factor in nervous system deterioration. Pain, divided into thermal, mechanical, and hyperalgesic types, is one of the most ill-defined symptoms of diabetic neuropathy. Scheffler et al. (154) studied OLE’s ability to impede Ca^2+^ channel activity. These calcium ions successfully treat pain without causing any adverse side effects by playing a physiological role in regulating pain sensitivity.

### Anticancer activity

The great epidemiological proof is that people who follow the Mediterranean diet have a lower risk of colon, skin, and breast cancers. The oxidation of lipids, proteins, and DNA has contributed to tumor growth. Including antioxidant components in the diet decreases the threat of carcinogenesis and mutagenesis (166). In large quantities, antioxidants and polyphenols in vegetables, olive fruits, and oil are constituents of the Mediterranean diet. *In vitro* examinations have noticed that olive oil polyphenols are effective antioxidants that might be delivered potential chemoprotective properties (167). Hydroxytyrosol is a phenolic compound that can mitigate the oxidative damage caused by H_2_O_2_ and peroxynitrite on cells and DNA. They hinder cell cycle advancement at the G1 phase and induce apoptosis. Oleuropein has also been associated with invasiveness, antiangiogenic action, motility, and inhibits cell growth. Breast cancer is a significant concern for women in developed countries (168, 169), and its occurrence has risen by more than 20% globally since 2008 (170). Etiological studies revealed that women residing in the Mediterranean region have a lower occurrence of breast tumors than in the USA and Scandinavian countries (171, 172).

Usually, this type of cancer is related to diet, mainly fewer fruits and vegetables and high meat consumption. At the same time, the diet of the Mediterranean basin includes a high amount of food from plant origins, a relatively lower quantity of red meat, and high consumption of olive-related products, which in numerous studies have been stated to be more helpful against cancer. Toledo et al. (170) provided a control Mediterranean diet supplemented with olive oil to women for 4.8 years. They observed a lower frequency of breast tumors in studied women than in control groups. High mammographic breast density (H-MBD) is linked with a higher prevalence of breast cancer. Keys et al. (171) examined 44 volunteers from the European Perspective Investigation into Cancer and Nutrition (EPIC) in their study, observed the influence of lifestyle and diet on MBD, and determined that intake of olive oil no relation to the hazard of H-MBD. Women with olive oil consumption of ≥30.5 g/day had 30% fewer occurrences in the H-MBD group.

Polyphenols and flavonoids from olive leaves and fruit extract have the strong antioxidant ability and anticancer effects (173–177). So, these extracts reduce the incidence of cervical carcinoma cells, colon cancer, and breast cancer (48). The polyphenols in OLE, i.e., hydroxytyrosol (HT) and oleuropein (OLP), significantly reduce breast cancer. It has also been studied to prevent cell apoptosis, decrease cell viability and proliferation, and considerably terminate the cell cycle in the growth 1 (G1) phase.

Milanizadeh et al. (178) transplanted tumors in mice and showed that consumption of OLE decreased the weight and volume of the transplanted tumor. Their highly packed polyphenols increased superoxide dismutase and catalase activity in tumor tissues. Carcinogenic compounds are produced by frying protein-containing food; these compounds induce pancreatic, breast, and colon cancer (179). In contrast, foods fried in olive oil shield against colon cancer because of the flavonoids, polyphenols of olive oil, and lesser carcinogenic compounds are produced during frying. Galeone et al. (180) collected data from a multinational and found a relationship between colorectal cancer and fried foods.

Furthermore, Gill et al. (168) analyzed the impact of olive phenols on the colorectal tumor. They studied the three main stages of cancer growth; initiation, promotion, and metastasis, and determined that olive phenols have appreciable effects in all three stages. Olive polyphenols have been demonstrated to decrease DNA damage (initiation), improve barrier properties (promotion), and decrease cell invasion of neighboring tissue (metastasis). Rheumatoid arthritis (RA) is characterized by chronic joint and tissue inflammation and damage. It is an autoimmune disease, but its primary stimulus is unknown. Tissue and joint inflammation arise by various mechanisms, mainly due to reactive oxygen species (ROS). Oxygen species can disrupt collagen, protease inhibitors, membrane function, hyaluronic acid, and proteoglycans due to the oxidation of membrane fatty acids (181, 182). The leading cause of RA is usually due to an elevation in the level of neutrophils and macrophages in the synovial fluid and enzymes that produce free radical species, which increase in joints and results in damage and inflammation (182).

Olive leaf extract has significant antioxidant activity and a substantial inhibitory impact on the growth of cancer and endothelial cells. In this study evaluating the effect of OLE on human breast adenocarcinoma (MCF-7), human urinary bladder carcinoma (T-24), and bovine brain capillary endothelial cells (BBCE), luteolin and its glucosides were the most active compounds, inhibiting cancer and endothelial cell proliferation at low micromolar concentrations (183).

The lower concentrations of oleuropein (0.005–0.025%) can inhibit the proliferation of human myeloid progenitor cells (TF-1a), human breast ductal carcinoma cells (T-47D), and human malignant melanoma cells (RPMI-751). However, renal adenocarcinoma cells (768-a) are inhibited at high concentrations of oleuropein. In addition to decreasing cell growth, oleuropein decreased cell motility and cell invasion *in vitro*. Additionally, oleuropein produced cell rounding, which led to the breakdown of the actin cytoskeleton. In this investigation, oleuropein disrupted both pure actin filaments and the actin cytoskeleton in live cells. These effects were reversible on normal cells but irreversible on cancer cells, showing that oleuropein acts selectively against normal and cancer cells. The removal of glucose from oleuropein decreased the inhibitory action, indicating a glucose-based entry pathway into the cell. It has been observed that certain human cancer cells, including those of the thyroid, prostate, cervix, breast, and colon, overexpress certain GLUTs. Therefore, cancer cells that over-express GLUTs may be more susceptible to oleuropein, which may explain the differential sensitivity of various cell lines (leukemia > melanoma > colon and breast > kidney). In addition, the fact that normal cells have no or minimal expression of some GLUTs may explain why oleuropein’s actions are reversible in normal cells but not in cancer cells. This study also investigated the antitumor activity of oleuropein *in vivo*. Using Swiss albino mice that naturally develop soft tissue sarcomas, oral administration of 1% oleuropein in the drinking water resulted in substantial regression of tumors.

Tumors larger than 2 cm in diameter regressed completely in 10 of 11 animals and partially in one. Oleuropein appeared to have the same impact on mice with single or numerous lesions, with several lesions regressing just slightly more slowly (9 vs. 12 days). This study also revealed that the tumors of oleuropein-treated mice were non-cohesive and crumbly, but those of untreated animals were more fibrous and solid (184). Oleuropein potentially represents a new class of anticancer drugs due to its antioxidant action, preventing mutations and genetic damage. Additionally, antiangiogenic activity prevents tumor development and its direct inhibition of cancer cells via tumor regression. Due to the unique combination of such actions in these chemicals, olive products and their derivatives should be transformed from dietary supplements into anticancer agents worthy of human testing (184).

### Cardiovascular activity

Extra virgin olive oil is a typical source of visible fat for people in the Mediterranean basin; thus, olive is a priceless and precious source of vitamins, monounsaturated and di-unsaturated fatty acids, and polyphenolic antioxidants (185). *Oleuropein* is the leading glycoside that has a beneficial impact on human health. OLE has a high phenolic content, which contains oleuropein, which inhibits lipoprotein oxidation (186, 187). Oleuropein has cardioprotective influence against hypolipidemic severe Adriamycin cardiotoxicity and an anti-ischemic activity (186, 188).

Oleuropein revealed neuroprotection by making a non-covalent composite through the Ab peptide, a crucial trademark of numerous degenerative disorders like Parkinson’s and Alzheimer’s (186, 189). It was stated that the fat quality is more important than the whole quantity utilized. The Mediterranean diet has a comparatively high-fat content but has a protective action against CVD and cancer (167). It was observed that OLE conquers the L-type calcium channel reversibly and directly; these channels have a direct part in CVD functions. Thus, OLE or other derived substances from olive fruit and leaves provide a source for developing novel beneficial drugs in curing cardiovascular and hypertension diseases (190).

Olive and its products (olive oil, fruit, OLE) have antihypertensive properties (191). Hidalgo et al. (192) studied how olive oil extract lowers the mean arterial systolic and diastolic blood pressures in normotensive rats (90, 193). Oleic acid and phenols reduce the ROS and thus can improve endothelial functions (90, 194). Oleuropein lowers the blood vessels’ tension and supports the vessels’ spread, finally dropping blood pressure (154). OLE combats endothelial dysfunction at numerous levels. They produce nitric oxide that assists in relaxing blood vessels (195).

Oleuropein decreases the activity and production of a class of molecules called matrix metalloproteinases (MMP). Undue MMP activity liquefies the gel-like matrix that clasps the cells together, creating vessel linings gradually vulnerable to plaque damage (110). Polyphenol composites present in olive plants prevent the formation of arterial plaques in two ways. First, they decrease the activity and production of a series of “adhesion molecules” (white blood cells and platelets) that cause plaque formation (196, 197). These substances stick to arterial walls, resulting in the formation of early plaque. Second, they decrease platelet aggregation, which decreases the possibility that small clots form at the plaque sites to cause a heart attack or stroke (198, 199). Constituents of the phenolic portion of olive inhibit eicosanoid formation and platelet function. An *in vitro* study on the blood of 11 healthy, non-smoking males showed that olive leaf polyphenols inhibit platelet function. OLE (at high levels of oleuropein) caused substantial dose-dependent destruction of platelet aggregation and platelet-ATP release (200).

### Neuroprotective effect

Aging is because of the oxidative injury of mitochondria throughout a lifetime, owing to the free radical concept. This oxidative damage is irreversible and causes cellular dysfunction (201). Mitochondrial membranes are very susceptible to free radical attacks because they have a double carbon-carbon bond in the lipid ends of their phospholipids. Hence, it causes the development of neurodegenerative and cognitive diseases. Several epidemiological and *in vitro* studies have found that extracted polyphenols reduce the occurrence of age-related ailments such as dementia (202).

In an investigation, Panza et al. (203) stated that oleuropein could reduce or even avoid the aggregation of Ab peptides, which is the leading cause of Alzheimer’s disease (AD). The possible influence of oleuropein on brain function has been stated. Alzheimer’s disease is similar to atherosclerosis because both are age-dependent diseases wherein the abnormal cholesterol deposition leads to symptoms, precedes disease, and becomes the link between hypercholesterolemia, AD, and heart disease (204–206). There is circumstantial evidence that cholesterol-related interventions can differ in Ab peptide deposition and oleuropein had an encouraging effect in managing AD (206). Additionally, polyphenols affect inflammatory reactions in the clinical expression of AD, as shown by epidemiological signs of the defensive effect of anti-inflammatory agents vs. AD. Thus, polyphenol extracts like oleuropein are effective vs. age-dependent pathologies (207–209).

## Conclusion

The scientific evidence confirms the health advantages of olive consumption, which encourages the opening new marketplaces that use olive waste to meet consumers’ health-related needs. According to the data gathered in this review, by-products derived from *O. europaea* L. The secondary valuable products from the processing industry have biologically active molecules that can be recovered and reused for food, pharmaceutical, and cosmetic uses through circular economy policies. One of the greatest benefits is using valuable components from olive by-products in functional meals. Olive leaves have been exploited for centuries for their different medicinal properties. Recent research has proven that OLE has several favorable implications for human health. Despite the vast research on OLE and its positive benefits, human studies on this topic are still extremely uncommon. Human studies have solely examined the skin-protective, hypoglycemic, and platelet aggregation effects of the research listed above. Even while some human studies have been conducted on the metabolism and bioavailability of OLE chemicals, the data are still inadequate. They cannot give a comprehensive understanding of this topic. The anticancer, hypoglycemic, anti-inflammatory, and cardiovascular effects of herbal compounds such as OLE are of particular interest due to the rising incidence of cancer and chronic diseases such as diabetes mellitus and cardiovascular diseases, as well as the growing need to find new treatments for these conditions. OLE’s cardiovascular, anti-inflammatory, and anticancer properties have previously demonstrated impressive results in *in vitro* and animal research; it is time to investigate these benefits in human trials. As a keystone for other benefits, the specific mechanism of action for antioxidant actions remains unknown. Uncertainty surrounds the extent to which these effects can apply to people. Regarding the antibacterial activities of OLE, which have only been investigated *in vitro*, more animal research may bring us one step closer to understanding these effects and employing them in future human trials. Although olive leaf and its components have a long way to go before, they can take their position among the medications actively utilized in contemporary medicine, their outstanding health-beneficial benefits are worthy of additional investment and research.

## Data availability statement

The datasets used and/or analyzed during the current study are available from the corresponding author on reasonable request.

## Author contributions

All authors were equal contributors in writing of this manuscript, read and approved the final manuscript.

## Footnotes

1
http://www.internationaloliveoil.org/
